# Accurate determination of post-operative 3D component positioning in total knee arthroplasty: the AURORA protocol

**DOI:** 10.1186/s13018-018-0957-0

**Published:** 2018-10-30

**Authors:** Edgar A Wakelin, Linda Tran, Joshua G Twiggs, Willy Theodore, Justin P Roe, Michael I Solomon, Brett A Fritsch, Brad P Miles

**Affiliations:** 1 360 Knee Systems, Suite 3, Building 1, Sydney, NSW 2073 Australia; 2 Department of Biomedical Engineering, University of Sydney, Sydney, NSW 2006 Australia; 3 North Sydney Orthopaedic and Sports Medicine Centre, The Mater Hospital, North Sydney, NSW 2065 Australia; 4 Prince of Wales Private Hospital, Barker Street, Kensington, NSW 2030 Australia; 5 Sydney Orthopaedic Research Institute, 445 Victoria Ave, Sydney, NSW 2067 Australia

**Keywords:** Registration, CT scan, Total knee arthroplasty, Alignment, Reliability, Reproducibility

## Abstract

**Background:**

Successful component alignment is a major metric of success in total knee arthroplasty. Component translational placement, however, is less well reported despite being shown to affect patient outcomes. CT scans and planar X-rays are routinely used to report alignment but do not report measurements as precisely or accurately as modern navigation systems can deliver, or with reference to the pre-operative anatomy.

**Methods:**

A method is presented here that utilises a CT scan obtained for pre-operative planning and a post-operative CT scan for analysis to recreate a computation model of the knee with patient-specific axes. This model is then used to determine the post-operative component position in 3D space.

**Results:**

Two subjects were investigated for reproducibility producing 12 sets of results. The maximum error using this technique was 0.9° ± 0.6° in rotation and 0.5 mm ± 0.3 mm in translation. Eleven subjects were investigated for reliability producing 22 sets of results. The intra-class correlation coefficient for each of the three axes of rotation and three primary resection planes was > 0.93 indicating excellent reliability.

**Conclusions:**

Routine use of this analysis will allow surgeons and engineers to better understand the effect of component alignment as well as the placement on outcome.

## Background

Dissatisfaction amongst total knee arthroplasty (TKA) is the result of a complex relationship between the patient anatomy, prosthesis design and position, and other patient-specific factors. Prosthesis malalignment has been linked to poor patient outcomes in which coronal and axial malalignment has been most closely studied [1, 2]. To have confidence in the correlation between component alignment and outcome, the method used to determine component placement must be accurate and reliable.

Component alignment refers to the angular difference between the prosthetic components and patient-derived antero-posterior (AP), medio-lateral (ML), and superior-inferior (SI) anatomic axes. This measurement has traditionally been the focus of post-operative analysis in TKA due to the ease of measurement [3–5]. Component placement refers to the translational movement of the prosthetic components along these patient-specific axes. Due to difficulty in identifying the origin of these axes and accurately determining translation in space, component placement has been less well investigated. To understand the holistic effect of the TKA components on knee kinematics, both the alignment and placement must be taken into account. Here, we term the combination of component alignment and placement as ‘component position’.

The pre-operative state of the patient is a critical source of missing data from most analyses which prevents accurate reporting of component position. Bony resections cannot be accurately determined from a post-op analysis alone, and as a result, there is very little data available on the outcome of TKA as a result of the modification of the anatomy [6, 7], highlighting the need for improved post-operative analysis techniques. Nevertheless, studies have investigated a range of movement and maximum flexion as a function of the posterior condylar offset (PCO) [8–10]. In these publications, a greater PCO resulted in higher maximum flexion due to reduced steric hindrance. Pre- and post-operative measurements however were limited by the use of ML X-rays, indicating that the relationship must be strong to overcome such errors.

Alteration of the joint line and flexion/extension gaps is associated with a change in joint kinematics [11] and patient outcome [12]. In these studies, patients with less change to the coronal joint line reported improved WOMAC and Knee Society Clinical Rating Scores. Identification of such changes however can be difficult, as the joint line and joint gaps can be modified without affecting the appearance of the component alignment [5]. To better understand the effect of bone resections and joint line and gap modification, accurate pre-operative geometry data is required. Similarly, Bengs and Scott [13] found that increasing the patella button thickness without increasing the patella resection decreased the maximum passive flexion. Identification of appropriate patella resection for a given button thickness would not be possible with traditional post-operative analysis techniques.

Traditional methods of assessing TKA component alignment, including short leg X-rays [14–16], long leg X-rays [17], and post-operative 3D imaging only [18–22], have been shown to suffer inaccuracies from anatomic variability and projection errors and difficulty in identifying patient-specific landmarks from the post-operative imaging. To improve landmarking and component placement accuracy, a pre-operative CT is required. Fortunately, CT imaging is rapidly becoming a standard of care in pre-operative planning for TKA [23] and is available for a wide range of patients. Pre-operative CT imaging allows a volumetric registration of the pre-operative and post-operative bones and component geometries in 3D space eliminating any anatomic assumptions and projection errors. The models can then be used to determine bony resections and component placement. A method to compare the pre-operative state of the knee to the post-operative component position and bone resections, in which accuracy has not been affected by component flare, has not yet been achieved.

Here, we introduce a method of 3D reconstruction which utilises both a pre-operative and post-operative CT scan to determine the post-operative component position in TKA. The method may be extended to any joint replacement and is termed here the Australian Universal Resection, Orientation, and Rotation Analysis (AURORA) protocol. Landmarks and bone models unaffected by component flare obtained from the pre-operative scan are transformed into the post-operative frame of reference. Component position as defined by the landmarked patient-specific axes and bony resections are reported. The reproducibility and reliability of this method are presented and compared to other post-operative analysis techniques.

## Methods

### CT protocol

**Fig. 1:**
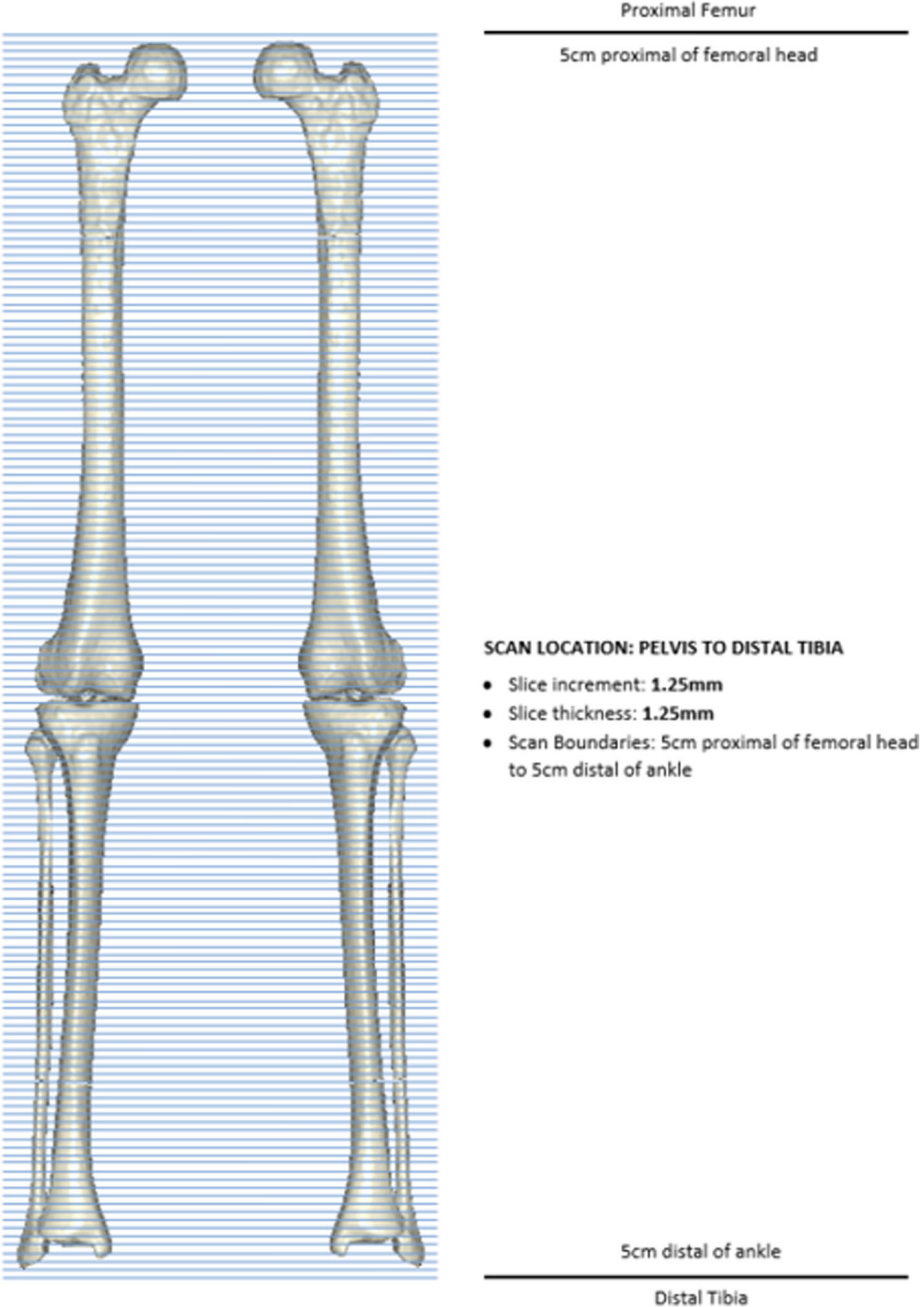
Single-pass CT scan through both limbs

A series of patients received long leg pre-operative CT scans for the routine pre-operative planning of TKA surgery [24] and to design patient-specific instrumentation. Ethics approval for all data collection and accessing information from a joint registry for this study was approved by Bellberry Ethics (Sydney, Australia) (approval 2012-03-710). The same protocol is followed for post-operative CT imaging. This protocol requires the patient to be in supine at the isocentre of the gantry, with both legs fully extended and parallel to the horizontal plane. The legs are straightened and maintained in a relaxed position. Image acquisition involves a full leg pass CT scan taken through both limbs with all images taken in the same field of view, see Fig. 1. This allows detection of any patient movement during the scanning process. Transverse slice thicknesses of 1.25 mm are taken, with less than 1 mm slices taken within the sagittal and coronal axis.

All patients investigated here had a TKA using OMNI APEX implants (Raynham, MA), from four different surgeons using four different techniques. Patients were randomly selected from a database of over 2000 TKA surgeries.

The CT dose is calculated by multiplying the dose-length-product (mGy.cm) provided as supporting information with the CT scan, by the length of the CT scan in which the patient is imaged. The dose value is then converted to an effective dose based on anatomic conversion coefficients presented by Saltybaeva et al. [25] to allow comparison between different CT protocols. Movement in the scan can affect both individual bone and long leg measurements. Movement is detected by an engineer assessing the scan before processing. All patients were randomly selected from a database of patients scanned over a 3-month period previously confirmed to have not moved.

### Image processing and volumetric registration

**Fig. 2:**
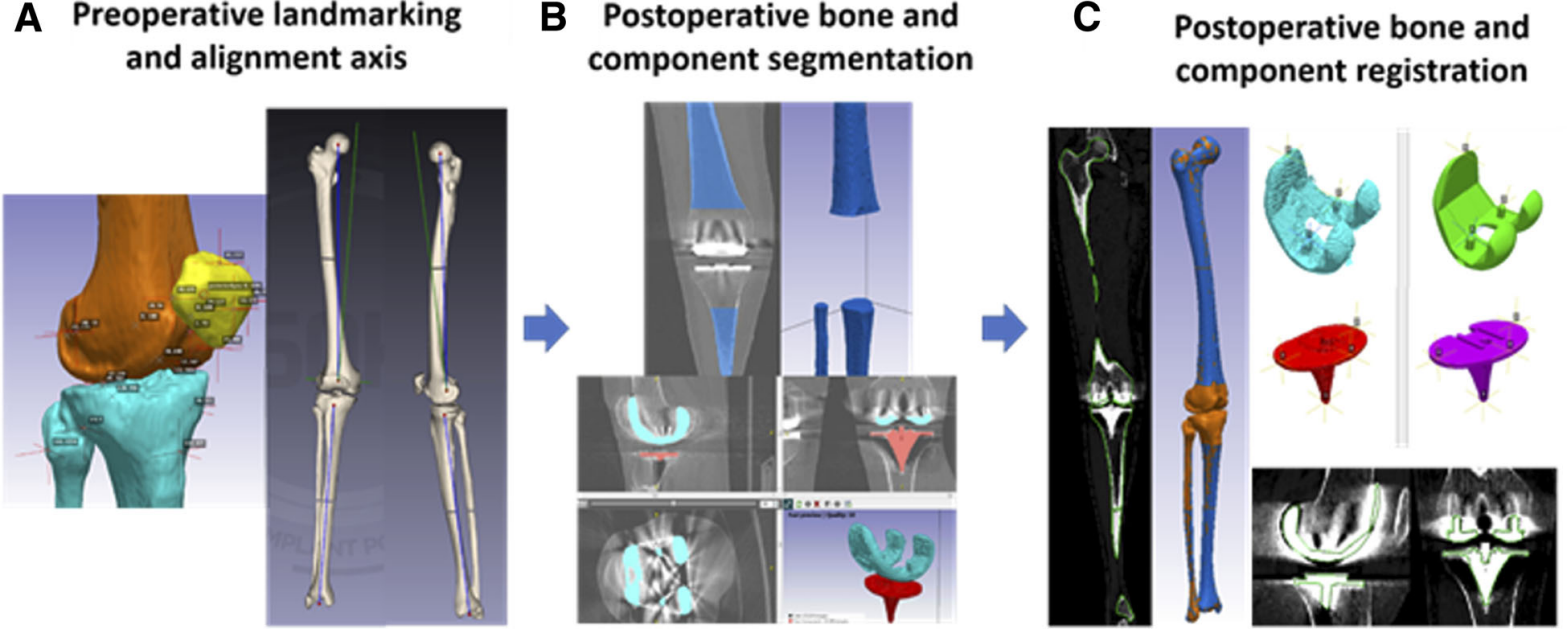
Post-operative process workflow showing (**a**) pre-operative bone segmentation and landmarking, (**b**) segmentation of post-operative bones and components, and (**c**) registration of pre-operative to post-operative bones and components

3D reconstructed patient femur and tibia bones are generated within the pre-operative planning process through semi-automated segmentation, used to landmark and identify points of interest by biomedical engineers using the 3D imaging software, ScanIP (Simpleware, Exeter, UK). The patient bones are converted to stereolithography (STL) files and landmarked twice by different engineers. If any landmarks differ by a threshold value (in this case 4 mm), the landmark was reviewed by another trained engineer. Landmark references were used to define patient-specific bone axes and soft tissue attachment sites, see Fig. 2a. The femoral and hip centres are landmarked to define the mechanical axis of the femur. The tibial mechanical axis is defined from the midpoint of the lateral and medial malleoli to the midpoint of the medial 1/3 of the tubercle and posterior cruciate ligament (PCL) insertion. The tibial AP axis is defined along the medial 1/3 of the tubercle and PCL insertion, while the transepicondylar axis (TEA) is defined along the medial sulcus to the lateral epicondyle on the femur. These axes are used to define a frame of reference from which implant position may be calculated.

Using the post-operative full leg CT scan, the 3D post-operative femur and tibia bone sections unaffected by the component flare are segmented, see Fig. 2b. 3D registration is then performed, by registering the pre-operative femur and tibia models into the post-operative CT with reference to both the imaging and newly generated post-operative bone models, see Fig. 2c. Point-to-point registration is performed on CAD models of the implanted prosthesis and segmented prosthesis models from the CT, see Fig. 2c. All registration is refined using model outlines viewed in the full leg CT scan. A second engineer reviews both the registered femur and tibia bones and the femoral and tibial implant components to further refine both bone and implant positions within the CT scan.

Euler transform matrices are obtained from the resulting registered pre-operative bones and used to transform the pre-operative bone landmarks into the post-operative CT reference frame. Using the transformed landmark references, component alignment and placement are determined within the local reference frames from the defined axes of landmarks identified pre-operatively.

### Accuracy testing

#### Reproducibility

**Fig. 3:**
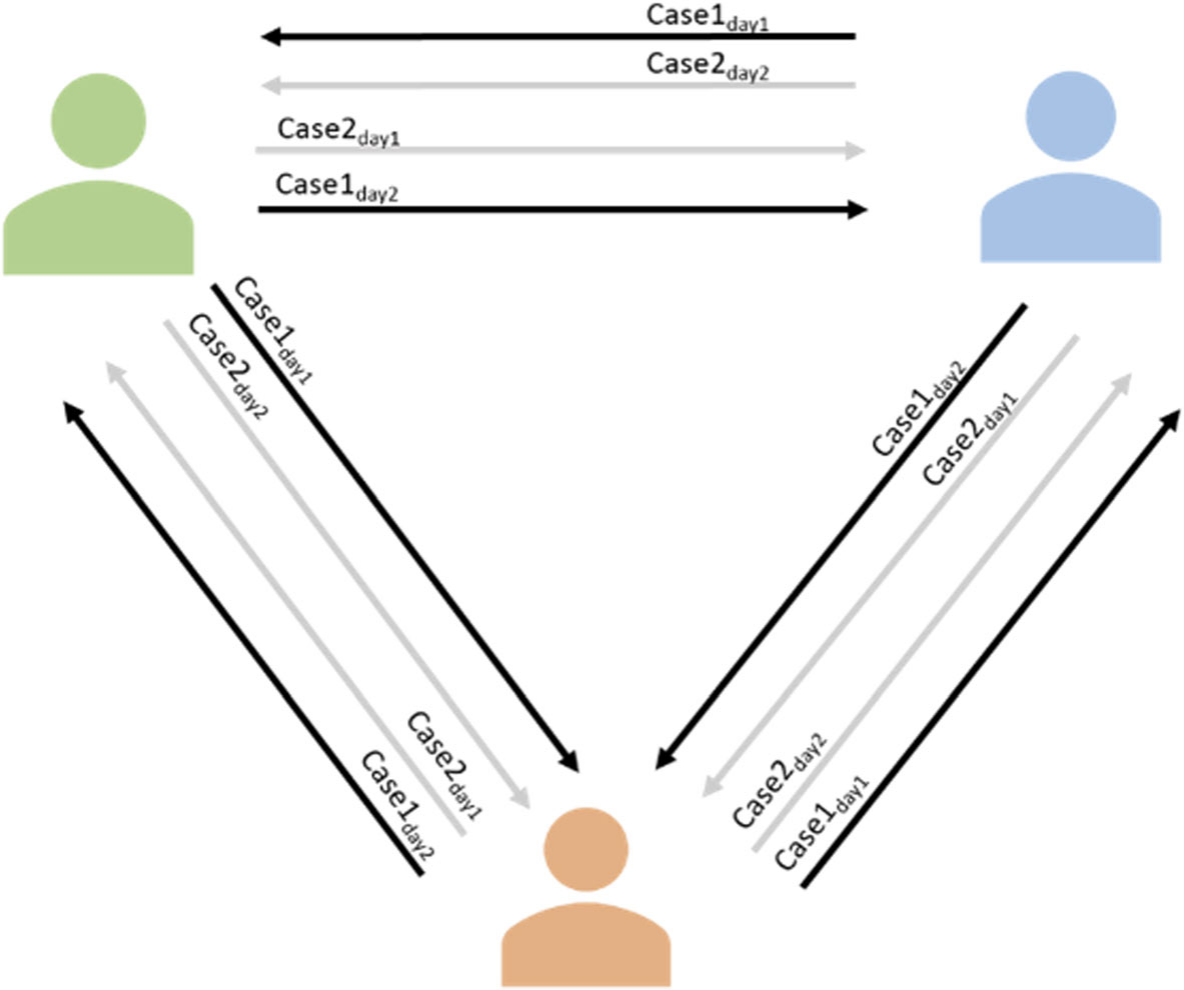
Flow diagram of reproducibility quality control

Two primary TKR patients were processed post-operatively twice by three engineers in a 2-week period. Patient CT scans were segmented and registered by an engineer and then reviewed by a second engineer. The same case was processed again by the initial engineer on another day at a different time of day and then reviewed by a third engineer. This process was repeated across the three engineers for the two cases with alternating reviewers, and a total of 12 registrations was then analysed (see Fig. 3).

Comparison of component alignment angles in flexion/extension (FE), varus/valgus (VV), and internal/external (IE) rotation, and component placement values by measuring the femoral medial and lateral, distal and posterior condyles, and the medial and lateral tibial plateau was recorded. Reproducibility was assessed from these angular and resection measurements by determining the maximum difference and standard deviation from the mean calculated for each patient, with the 95% confidence interval defined across both cases.

#### Reliability

To describe the interobserver reliability, 11 TKR patients were processed post-operatively between two engineers. Each case was reviewed by a third and fourth engineer, with refinement of the bone and component registration made by the reviewing engineer if necessary. A set of 22 results was produced for the comparison of the three rotation axes across two components and six resection measurements. The intra-class correlation coefficient (ICC) was calculated for each of the measurements. An ICC value of 1 shows perfect reliability, values greater than 0.9 indicates an excellent result, 0.81 to 0.9 is very good, 0.76 to 0.80 is good, 0.5 to 0.75 is moderate, and < 0.50 is considered to show poor reliability [26, 27].

## Results

### Radiation dose

**Fig. 4:**
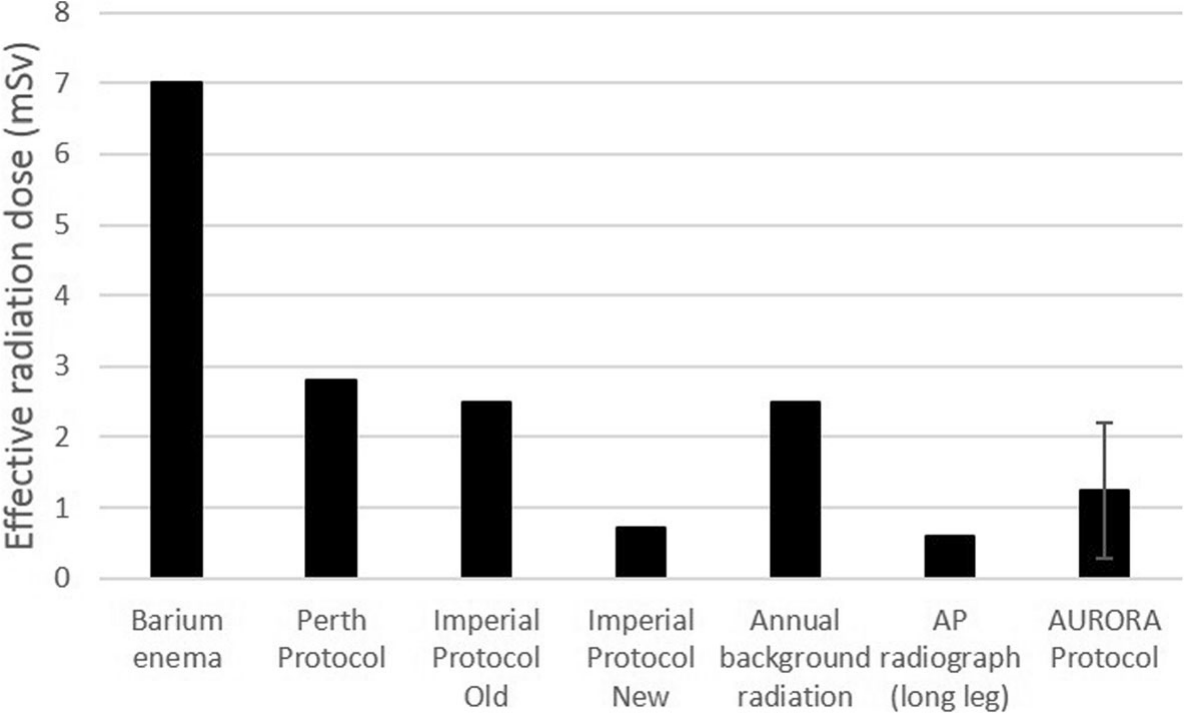
Comparison of the AURORA CT protocol with a barium enema and other relevant protocols for determining prosthesis positioning. AURORA protocol dose is calculated from CT reports, and all other data taken from Henckel et al. [6]

The average effective radiation dose received per CT scan using this protocol is 1.24 ± 0.96 mSv. This dose is compared to other CT and radiography protocols in Fig. 4. The average received dose is lower than all protocols shown in the figure with the exception of the most recent Imperial Protocol [6] and a standard AP radiograph. The spread of values shown for the AURORA CT protocol used here reflects the large range of patient sizes scanned. Smaller patients receive a correspondingly lower dose of radiation and vice versa for larger patients.

Using the AURORA protocol, patient movement in the CT scan may be detected at any point along the length of the bone. In previous methods, such as the Perth CT and Imperial protocols, movement in the mid-femur and mid-tibia will not be detected, leaving any measurements to propagate through the protocol as an error. In a database of CT scans obtained for the routine pre-operative planning of TKA, the rate of scans identified with movement over a 3-month period is 6.78% (total number of scans, 118). Of this fraction, all movement in the scans were detected in the mid-femur and mid-tibia regions.

### Reproducibility

The alignment reproducibility results generated from three engineers processing two cases at two different time points which were then QC checked are shown in Table 1. The maximum difference from the mean angle is shown for each case. In both cases, the maximum difference is reported for tibial component axial rotation, of 0.9° for case 1 and 0.7° for case 2. In all other angles, the maximum difference in rotation is ≤ 0.5°. The confidence intervals in all cases are less than 0.3° with the exception of tibial tray IE rotation, which is 0.6° for case 1 and 0.4° for case 2.

**Table 1:** Reproducibility results showing the difference in calculated component angular alignment across two cases performed by three engineers at two different time points. The maximum average difference for each case and a 95% confidence interval are shown for all three axes of rotation for the femoral and tibial components

Case	Operator	Run	Femoral component alignment			Tibial component alignment		
			F/E	V/V	IE	F/E	V/V	IE
Case 1	Sim Eng A	Run 1	1.1	− 0.6	− 0.8	7.1	0.6	5.9
Case 1	Sim Eng A	Run 2	1.0	− 0.5	− 1.2	7.0	0.4	6.2
Case 1	Sim Eng B	Run 1	1.0	− 0.8	− 1.5	7.0	0.5	4.5
Case 1	Sim Eng B	Run 2	0.8	− 0.7	− 0.8	7.4	0.4	4.7
Case 1	Sim Eng C	Run 1	1.1	− 0.6	− 1.8	6.9	0.8	5.5
Case 1	Sim Eng C	Run 2	1.1	− 0.4	− 1.0	7.2	0.6	5.9
**Maximum difference from average (°) ± 95% CI**			**0.2 ± 0.1**	**0.2 ± 0.1**	**0.5 ± 0.3**	**0.3 ± 0.1**	**0.2 ± 0.1**	**0.9 ± 0.6**
Case 2	Sim Eng A	Run 1	1.9	− 0.9	0.1	8.2	1.5	3.0
Case 2	Sim Eng A	Run 2	2.3	− 0.8	− 0.1	7.7	1.8	2.7
Case 2	Sim Eng B	Run 1	2.3	− 0.7	− 0.2	7.9	1.7	1.7
Case 2	Sim Eng B	Run 2	2.1	− 0.8	0.4	8.4	1.4	2.8
Case 2	Sim Eng C	Run 1	2.0	− 0.7	− 0.2	7.9	1.0	2.3
Case 2	Sim Eng C	Run 2	1.8	− 0.8	0.0	8.4	1.5	2.8
**Maximum difference from average (°) ± 95% CI**			**0.3 ± 0.2**	**0.1 ± 0.1**	**0.3 ± 0.2**	**0.3 ± 0.2**	**0.4 ± 0.2**	**0.7 ± 0.4**

The bony resection thicknesses are a proxy measure for the accuracy of measuring component placement and are shown in Table 2 for the distal medial and lateral condyles, posterior medial and lateral condyles, and tibial medial and lateral plateaus. The maximum difference from the mean resection is shown for each case. In both cases, the maximum difference is reported for the medial tibial plateau, of 0.5 mm for case 1 and 0.3 mm for case 2. In all other resections, the maximum difference in resection is ≤ 0.3 mm. The confidence intervals in all cases are less than 0.3 mm.

**Table 2:** Reproducibility results showing the difference in calculated bony resection thicknesses (giving a measure of the accuracy of component placement) for the distal medial and lateral condyles, posterior medial and lateral condyles, and tibial medial and lateral plateaus across two cases performed by three engineers at two different time points. The maximum average difference for each case and a 95% confidence interval are shown for all resections

Case	Operator	Run	Femoral resections				Tibial resections	
			Lat. condyle	Med. condyle	Post. lat. condyle	Post. med. condyle	Lat. plateau	Med. plateau
Case 1	Sim Eng A	Run 1	6.5	6.0	10.0	10.3	11.2	10.0
Case 1	Sim Eng A	Run 2	6.2	5.7	10.0	10.7	10.3	9.0
Case 1	Sim Eng B	Run 1	6.1	5.4	10.2	11.1	10.5	9.3
Case 1	Sim Eng B	Run 2	6.8	6.1	10.2	10.6	11.1	9.8
Case 1	Sim Eng C	Run 1	6.2	5.7	9.5	10.7	10.7	9.6
Case 1	Sim Eng C	Run 2	6.4	6.0	9.9	10.3	11.1	9.9
**Maximum difference from average (mm) ± 95% CI**			**0.3 ± 0.2**	**0.4 ± 0.2**	**0.4 ± 0.2**	**0.4 ± 0.2**	**0.4 ± 0.2**	**0.5 ± 0.3**
Case 2	Sim Eng A	Run 1	6.0	7.4	10.9	10.3	11.0	6.7
Case 2	Sim Eng A	Run 2	6.2	7.8	10.8	10.3	11.1	7.0
Case 2	Sim Eng B	Run 1	6.1	7.7	10.6	10.2	10.9	6.7
Case 2	Sim Eng B	Run 2	6.1	7.6	10.8	9.9	11.2	6.9
Case 2	Sim Eng C	Run 1	5.9	7.5	11.0	10.6	11.0	6.3
Case 2	Sim Eng C	Run 2	6.0	7.4	10.9	10.4	10.9	6.6
**Maximum difference from average (mm) ± 95% CI**			**0.2 ± 0.1**	**0.2 ± 0.1**	**0.2 ± 0.1**	**0.2 ± 0.1**	**0.2 ± 0.1**	**0.3 ± 0.2**

### Reliability

**Fig. 5:**
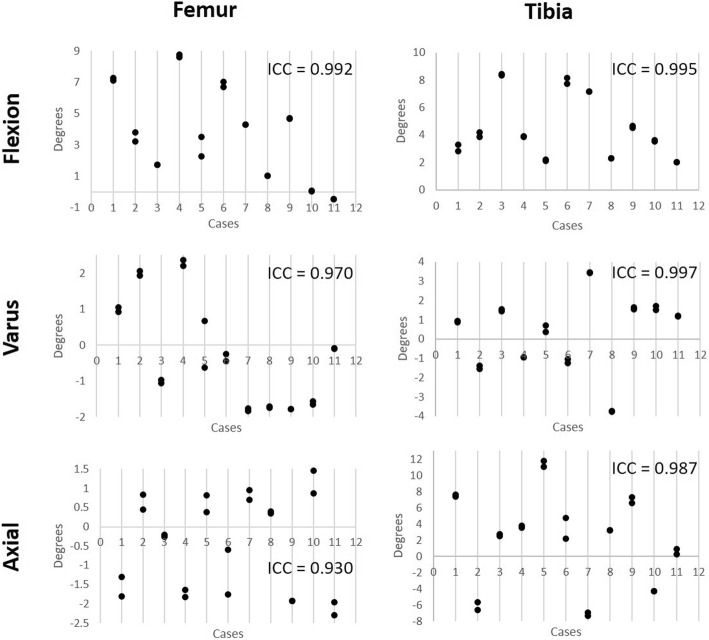
Reliability testing for femur and tibia placement showing the coronal, axial, and sagittal rotation reported by the method across 11 cases performed by two engineers, followed by two additional engineers reviewing the placement. The ICC for each rotation in each component is reported. All values are greater than 0.9 indicating excellent reliability

**Fig. 6:**
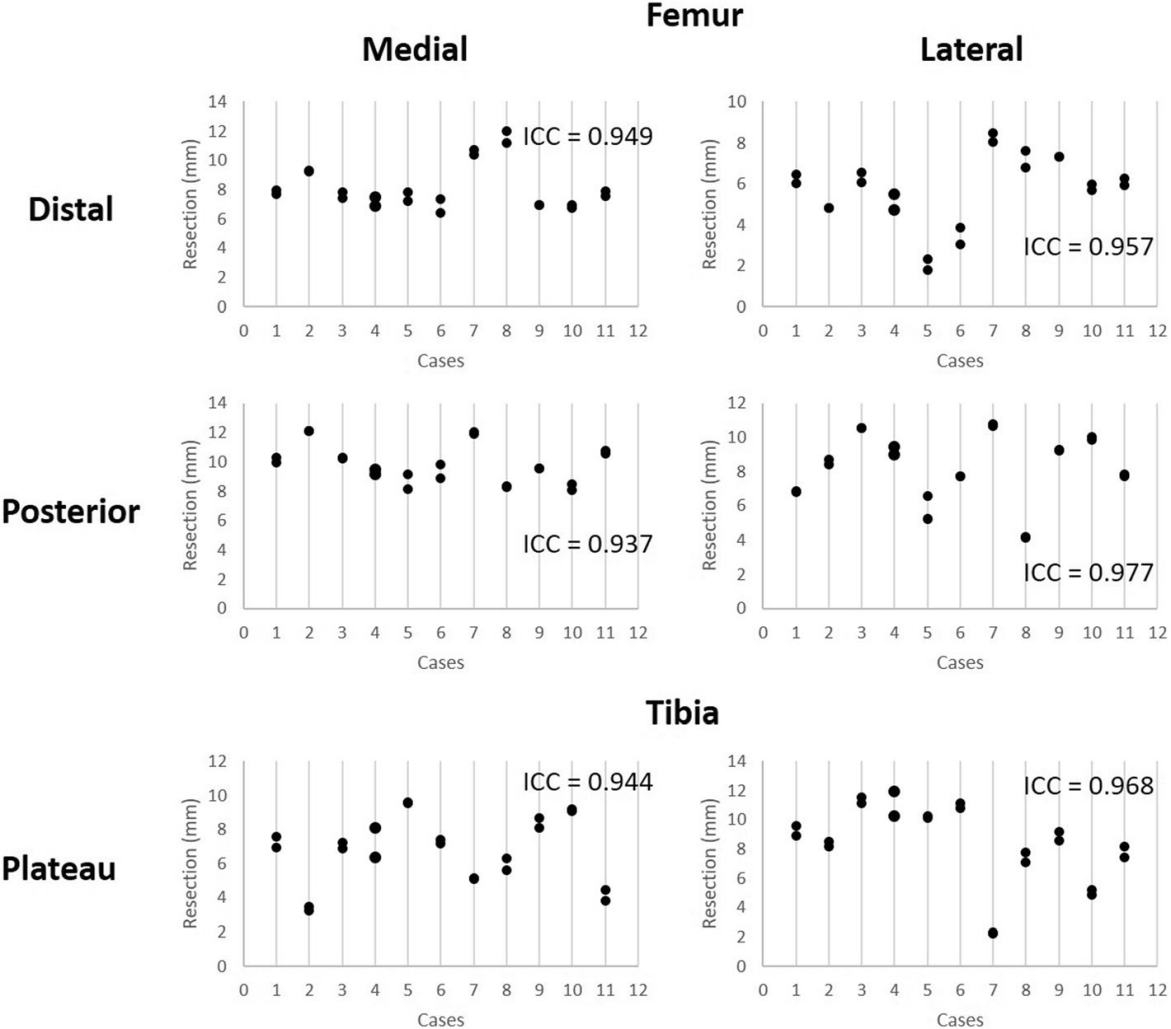
Reliability testing for femur and tibia bony resections reported by the method across 11 cases performed by two engineers, followed by two additional engineers reviewing the placement. The ICC for each rotation in each component is reported. All values are greater than 0.9 indicating excellent reliability

The rotational alignments and bony resections for the femur and tibial components reported for 11 cases performed twice (each time by a team of two different engineers) are shown in Figs. 5 and 6. The ICC value is given for each alignment and resection variable. The lowest reported ICC variable is for femoral axial rotation, with an ICC of 0.93. These values are all above 0.9, indicating that across all rotations and resections in both the femur and tibia, the protocol reports excellent reliability.

## Discussion

The maximum component alignment differences from the mean within this study are low compared to previous literature and provide a confidence interval up to tenfold narrower when compared to protocols in which individual CT slices were investigated [18, 19, 22, 28], or only post-operative CT scans were available [29, 30]. The maximum error of < 1° is similar to the protocols using more advanced techniques, such a computational edge detection; however, these studies did not include ICC coefficients, so an assessment of the repeatability was not possible [31]. The highest deviation from the mean was the tibial IE rotation at 0.9° and 0.7° for the two cases, with a confidence interval of 0.6° and 0.4°, respectively. These values represent an eightfold improvement in accuracy compared to previous attempts to measure tibial rotation [32]. Previous attempts have reported difficulty in measuring tibial IE rotation due to the variability in the landmarks required to define a useful axis [33]. By combining the pre-op and post-op CT, the landmarks that define the AP axis can be identified more easily than using post-op CTs alone. Although there may still be some debate over which landmarks are the most appropriate, this method allows points to be defined that accurately reproduce an anatomic axis across multiple subjects. The origin of all axes may be redefined based on future literature if needed.

The resulting resection level measures of the femur and tibia also show high reproducibility, with the highest deviation seen for the medial tibial plateau resection at 0.5 mm and 0.3 mm between the two cases and confidence intervals of 0.6° and 0.4°, respectively. The magnitude of the error here, however, is only slightly above the other resections, indicating that there may not be a systematic reason for reduced accuracy when placing this component. Previous attempts have been made to investigate the effect on TKA outcome arising from resection levels. These studies have mainly focussed on the femur, particularly the posterior condylar offset [8, 34, 35]. These techniques however have primarily relied upon fluoroscopic images and planar X-rays which were discussed previously to be inaccurate, limiting the reliability of such studies.

Across both femoral and tibial component alignments and bony resections, this 3D pre-operative registration process shows excelled reliability, in which all ICC values report greater than 0.93. The lowest reported ICC value of 0.93, resulting from the femoral axial IE rotation measure, is primarily due to the difficulty of post-operative registration of the femur component. The posterior condyles, which dominate the axial rotation positioning, of the APEX implant used in this study are thicker than the distal condyles (11 mm vs 9 mm) and tibial tray (~ 3 mm). As such, the CT flare is greater in these regions, reducing the accuracy of the registration. The ICC values reported here are consistently similar to or higher than other post-operative analysis techniques [7, 22, 36] indicating this method is not only accurate but suitable for routine post-processing by multiple users.

The high reproducibility and reliability of calculating both component alignment and bony resections performed by surgeons can lead to a better understanding of the influences of component alignment and component placement. The current literature has thoroughly reviewed the influence of component malalignment and poor patient outcomes [37–39]. Missing from all of these analyses, however, is an understanding of the patient’s pre-operative anatomy, leading Hadi et al. [37] to conclude that there is a dubious link between component malalignment and patient outcomes. From this post-operative analysis, we can begin to determine how the bony resections and the combination of component placement and alignment influence the outcome on a patient-specific level in greater detail. For example, the use of reliable bone resection measures from pre-operative bones may provide insight into the change of a patient’s soft tissue profile post-surgery. From the pre-operative CT scans, comparative ligament lengths and change in length resulting from component alignment and placement can be investigated from landmarked attachment sites. CT scans in this analysis, however, are performed in a non-functional supine position, such that the distance between ligament attachment sites may not be representative of the functional length of the ligament. Functional imaging may be introduced to this workflow in the future to this issue without a change in post-processing techniques.

The proposed 3D registration process for post-operative analysis involves additional pre-operative CT imaging compared to other processes [6, 18]. Though this increases X-ray exposure to the patient, pre-operative planning, generally requiring a CT scan, is becoming the standard of care for TKA [23], such that the pre-operative scans are not for post-operative analysis alone. The protocol used here is a low-dose CT, with radiation exposure less than the typical yearly background radiation and similar to protocols currently in use [6]. All patient movement identified in pre-operative scans occurred in the mid-femur and mid-tibia regions, indicating that protocols which did not include the mid-femur and tibia sections would report inaccurate component placement. The resulting error in component position if these scans were used is the subject of further study.

Manual translation and rotation of the pre-operative bones and component geometries into the post-operative CT scan is reasonably labour intensive, requiring on average 60 min to complete, before the registration is quality control checked by a second engineer with additional experience. Further refinement of the proposed post-operative analysis process could include the use of an automated registration method. A preliminary automated registration process using the iterative closest point (ICP) method [30] was performed on these cases. The registration time was observed to reduce to approximately 2 min, from which the results were then fine-tuned by one engineer and quality control checked by a second engineer, representing a 30-fold decrease in time. Further development of the ICP method to optimise parameters around fitting regions of interest, reliability, and time for analysis may allow accurate post-operative analysis to be part of routine care and is the subject of future studies.

Joint infection and component loosening are a cause of dissatisfaction and revision surgery. Joint infection can be identified by swelling of the joint and pathology reports; however, these are not always conclusive. Combining component position as determined using the AURORA protocol with SPECT imaging could identify bone metabolism associated with infection or component movement [40]. Although current methods integrating SPECT imaging with CT do not improve the accuracy of determining component placement, such methods may be used to augment a pre-operative and post-operative CT 3D reconstruction to add metabolic activity.

The proposed post-operative 3D registration method described here has some limitations. The current time taken for this analysis as mentioned is approximately 60 min; this represents a high engineering burden and must be reduced to improve use in routine analysis. Commercially, TKA component geometry varies between medical device manufacturers, forming a significant part of their IP portfolio, as such, the component geometries must be obtained from the implant companies, which may be difficult—limiting the generalisability of this technique to engineering firms with a close relationship with implant companies. The reproducibility analysis performed here utilises two cases processed at multiple time points by multiple engineers of equal training. To better understand the reproducibility, particularly when processing outlier or severely pathological anatomy, a greater number of cases should be analysed.

Other methods to assess component position such as bi-planar X-rays followed by 2D to 3D registration offer a number of advantages over a CT, such as providing long leg assessments in a functional state [41]. Such techniques, however, may require fluoroscopic agents [42] and may only capture the region around the knee and are performed on apparatus less widely available than a traditional X-ray or CT machines, limiting its use [31].

## Conclusion

Component alignment has been of great interest in total knee arthroplasty; however, the focus has previously been on achieved component alignment and identification of malalignment without regard for the component placement or pre-operative anatomy. The method presented here uses a low-dose CT scan to analyse the position and rotation of all components in 3D space, with a comparison to the pre-operative anatomy, allowing surgical changes to the joint to be determined. The method shows excellent reliability and reproducibility by removing the sources of error that are typically associated with post-operative total knee arthroplasty analysis. Routine use of this analysis in TKA as well as other joint replacement procedures will allow surgeons and engineers to better understand the effect of component alignment as well as the placement on outcome.

## Abbreviations

3D: 3-Dimesional
AP: Antero-posterior
AURORA: Australian Universal Resection Orientation and Rotation Analysis
CT: Computed tomography
FE: Flexion-extension
ICC: Intra-class correlation coefficient
IE: Internal-external
ML: Medio-lateral
PCO: Posterior condylar offset
SI: Superior-inferior
STL: Stereolithographic language
TEA: Transepicondylar axis
TKA: Total knee arthroplasty
VV: Varus/valgus
